# *In Vivo* Tumor Growth Inhibition and Antiangiogenic Effect of Cyclic NGR Peptide-Daunorubicin Conjugates Developed for Targeted Drug Delivery

**DOI:** 10.1007/s12253-019-00773-3

**Published:** 2019-12-09

**Authors:** Andrea Angelo Pierluigi Tripodi, Ivan Ranđelović, Beáta Biri-Kovács, Bálint Szeder, Gábor Mező, József Tóvári

**Affiliations:** 1 MTA-ELTE Research Group of Peptide Chemistry, Hungarian Academy of Sciences, Eötvös Loránd University, Budapest, Hungary; 2 Faculty of Science, Institute of Chemistry, Eötvös Loránd University, Budapest, Hungary; 3 Department of Experimental Pharmacology, National Institute of Oncology, Budapest, Hungary; 4 Research Centre for Natural Sciences, Institute of Enzymology, Hungarian Academy of Sciences, Budapest, Hungary

**Keywords:** Targeted tumor therapy, NGR peptides, Tumor growth inhibition, Antiangiogenic effect, CD13, Metastasis

## Abstract

Among various homing devices, peptides containing the NGR tripeptide sequence represent a promising approach to selectively recognize CD13 receptor isoforms on the surface of tumor cells. They have been successfully used for the delivery of various chemotherapeutic drugs to tumor vessels. Here, we report on the murine plasma stability, in vitro and *in vivo* antitumor activity of our recently described bioconjugates containing daunorubicin as payload. Furthermore, CD13 expression of KS Kaposi’s Sarcoma cell line and HT-29 human colon carcinoma cell line was investigated. Flow cytometry studies confirm the fast cellular uptake resulting in the rapid delivery of the active metabolite Dau = Aoa-Gly-OH to tumor cells. The increased in vitro antitumor effect might be explained by the faster rearrangement from NGR to *iso*DGR in case of conjugate **2** (Dau = Aoa-GFLGK(c[NleNGRE]-GG)-NH_2_) in comparison with conjugate **1** (Dau = Aoa-GFLGK(c[KNGRE]-GG)-NH_2_). Nevertheless, results indicated that both conjugates showed significant effect on inhibition of proliferation in the primary tumor and also on blood vessel formation making them a potential candidate for targeting angiogenesis processes in tumors where CD13 and integrins are involved.

## Introduction

**Fig. 1:**
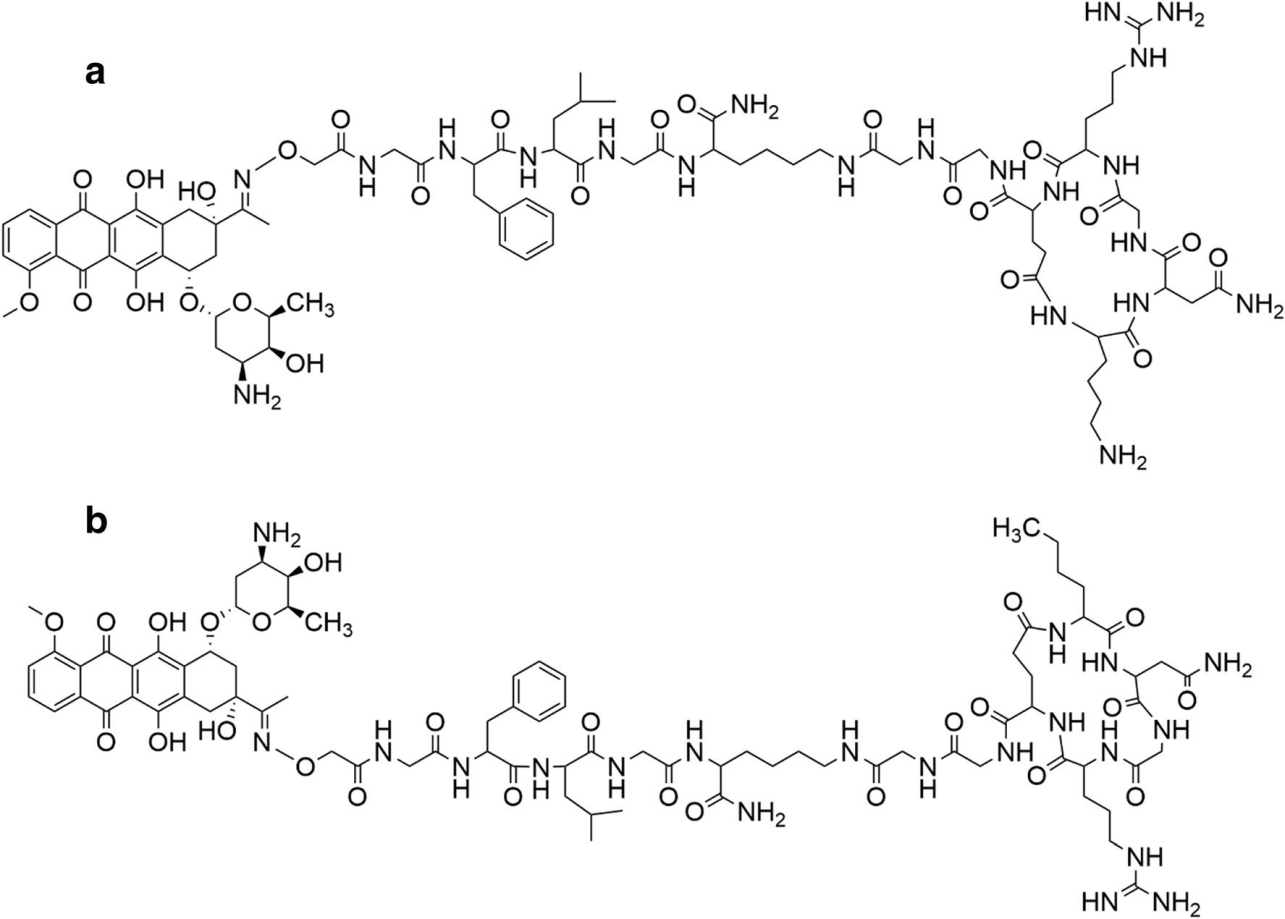
Structures of peptide-drug conjugates, Lys containing compound **1** (**a**) and Nle containing compound **2** (**b**)

In the last decades, huge efforts have been made to develop new strategies for the improvement of drug delivery and penetration into tumors [1–3]. Targeted tumor therapy is one of the most promising approaches that may provide a real breakthrough in this field [4]. The procedure is based on the different cell surface proteins and structures between healthy and tumor cells. Tumor specific antigens or overexpressed receptors on tumor cells can be good target molecules for selective drug delivery. Several antibody – drug conjugates (ADCs) are on market to treat different tumors [5, 6]. However, peptides as homing devices recently received increased attention in comparison with antibodies, due to their favorable pharmacokinetic properties for the targeted delivery of cytotoxic agents [7]. Cancer cells express a large number of receptors on their surfaces, some of them are overexpressed and mediate important biological functions in migration, invasion, tumor growth and metastasis [1, 8, 9]. For several years, many researches have been conducted to identify molecules that interact with receptors expressed in angiogenic vessels [10–15] that play a crucial role in tumor progression. Consequently, two important targets have been found on the new tumor vasculature; integrins and Aminopeptidase N (APN or CD13) [16]. CD13 is an ectoenzyme of 150-240 kDa [17–20] belonging to the family of zinc metallopeptidases with numerous functions: regulation of hormones and cytokines, cell proliferation, migration and invasion [21]. CD13 is expressed by many cells of normal tissue, including epithelial cells from small intestine, prostate, bile duct canaliculi and myeloid cells while it is up-regulated in angiogenic blood vessels and, in some cases by fibroblasts [20, 22–29]. Several peptides containing the NGR tripeptide motif that specifically recognize the CD13 receptor isoform on tumor cells have been successfully used for the delivery of various compounds and chemotherapeutic drugs to tumor vessels [30–37]. Peptides containing the cyclic CNGRC and linear GNGRG motifs have been applied for targeting tumor necrosis factor alpha (TNF-α) and interferon gamma [38–41]. To improve its therapeutic index, chemotherapeutic drug like daunorubicin as payload was attached to an NGR-peptide, and the resulted conjugate showed improved efficacy on tumor growth inhibition in mice with decreased peripheral toxicity in comparison with the free drug [11, 42]. Next to daunorubicin, other drugs such as platinum IV, carboplatin and 5-fluorouracil were coupled to NGR peptides as well [43–45]. However, it is well known that the NGR motif can easily undergo Asn deamidation through succinimide formation followed by hydrolysis that can occur at both carbonyl groups of the cyclic imide leading to the formation of Asp and *iso*Asp residues with a usual ratio of 1:3 [46–49]. The speed of this process is highly influenced by numerous factors like peptide structure, temperature or pH of the solution. The most important one is the presence of a Gly that follows the Asn in the sequence that can promote the reaction due to the lack of steric hindrance [50]. One of the most significant pharmacological consequences of this rearrangement is the loss of CD13 affinity. Therefore, Negussie et al. developed an amide bond containing head-to-side-chain cyclic NGR peptide c[KNGRE]-NH_2_ that showed enhanced stability against deamidation. Furthermore, the ε-amino group of Lys as a conjugation site could be easily used for the attachment of Oregon Green fluorescent label without modifying the recognition by CD13 [51]. Recently published data demonstrated that c[KNGRE]-NH_2_ could be successfully used for tumor diagnostic studies by PET, showing its specific binding to tumor tissues expressing CD13 receptors [31, 52]. Nevertheless, the rearrangement of NGR peptides to *iso*DGR derivative might provide a benefit too, because this compound can mimic the RGD motif, and can bind to α_v_β_3,_ α_v_β_5,_ α_v_β_6,_ α_v_β_8_ and α_5_β_1_ integrins with high affinity [53–56]. Thus, the deamidation process might result in NGR peptide-drug conjugates with dual-receptor targeting activity on both CD13 and integrins. Previously, we reported the synthesis of cyclic NGR peptides (with amide, disulfide or thioether bond in the cycle) where daunorubicin (Dau) was attached to the homing peptide via oxime-linkage through an aminooxyacetylated (Aoa) Cathepsin B cleavable GFLG spacer [37, 57]. Cathepsins are highly up-regulated in numerous tumors allowing the selective release of the active metabolite Dau = Aoa-Gly-OH which can bind to the DNA and inhibit cell proliferation. It was indicated that the novel conjugates had a good *in vitro* antitumor effect on the selected cell lines. Moreover, the biological activity of the compounds was also evaluated using both CD13(+) KS (Kaposi’s Sarcoma) cells and CD13(−) (but integrin receptor positive) HT-29 human colon adenocarcinoma cells [58]. It has been established that the toxicity and selectivity is greatly influenced by structure, internalization capability and propensity to deamidation [57]. In particular, compound **1** KNGRE (Dau = Aoa-GFLGK(c[KNGRE]-GG-)-NH_2_) and compound **2** NleNGRE (Dau = Aoa-GFLGK(c[NleNGRE]-GG)-NH_2_) (Fig. 1) drug-conjugates showed high antitumor effect and even their stability against deamidation was significantly different [57]. The latter was more sensitive to rearrangement than the KNGRE version which had high stability under the experimental conditions. It has to be highlighted that Nle has the same linear hydrocarbon chain without amino group at the end of the side chain compared to Lys. Because the relevance of in vitro antitumor effect is quite low in case of antiangiogenic conjugates, we decided to use these two lead compounds for further *in vivo* experiments. Our goal was to better understand how these highly similar peptides can influence the *in vivo* antitumor effect, keeping in mind the dual-targeting approach especially in the case of Nle containing peptide conjugate. Therefore, in our current study we investigated the *in vivo* antitumor activity of the two peptide-drug conjugates and their ability to inhibit the tumor growth and the formation of blood vessels in orthotopic colon cancer bearing mice. In addition, we performed in vitro and *in vivo* experiments on CD13(+) Kaposi’s Sarcoma (KS) cell line [59–61] to compare the targeting effect of conjugates. In order to clarify the background of their activity, proliferation index and ex vivo blood vessel formation was evaluated on every tumor models.

## Materials and Methods

### Chemical Reagents

Fmoc-Rink-Amide MBHA resin, 1-hydroxybenzotriazole hydrate (HOBt), 1,8-diazabicyclo[5.4.0]undec-7-ene (DBU), *N,N′*-diisopropylcarbodiimide (DIC), triisopropylsilane (TIS), piperidine, trifluoroacetic acid (TFA), diisopropylethylamine (DIPEA), and ninhydrin were purchased from Sigma-Aldrich Kft (Budapest, Hungary). Daunorubicin hydrochloride was provided from IVAX (Budapest, Hungary). *N,N*-dimethylformamide (DMF), dichloromethane (DCM) and diethyl ether (Et_2_O) were delivered by Molar Chemicals Kft (Budapest, Hungary). All the amino acid derivatives used for the preparation of the conjugates were obtained from Merck KGaA (Darmstadt, Germany) or Iris Biotech GmbH (Marktredwitz, Germany) with the highest available purity.

### Preparation of Cyclic NGR Peptide-Daunorubicin Conjugates

The cyclic NGR peptide-daunorubicin conjugates were prepared by a combination of solid phase peptide synthesis and chemoselective ligation (oxime bond formation) in solution as described in Tripodi et al. [57]. The crude peptides and conjugates were purified on a KNAUER 2501 HPLC system (KNAUER, Bad Homburg, Germany) was applied with a semi-preparative Phenomenex Luna C18 column (250 mm × 21.2 mm) with 10 μm silica (100 Å pore size) (Torrance CA). Linear gradient elution (0 min 5% B; 60 min 90% B) with eluent A (0.1% TFA in water) and eluent B (0.1% TFA in MeCN-H_2_O (80:20, *v*/v)) was used at a flow rate of 4 mL/min. The resulting fractions were lyophilized. Electrospray Ionization (ESI)-mass spectrometric analyses were carried out on an Esquire 3000+ ion trap mass spectrometer (Bruker Daltonics, Bremen, Germany). The freeze-dried bioconjugates were directly used for the in vitro and *in vivo* studies.

### Stability in Murine Plasma

NGR-Dau conjugates were dissolved in ddH_2_O, murine plasma was added, the obtained final concentration of the conjugates was 10 μM. Samples were incubated at 37 °C, and aliquots were taken at 0.5, 1, 2, 4, and 8 h. The experiment was concluded by addition of 10 μL pure acetic acid. The low molecular weight samples were analyzed by LC-MS, while the high molecular weight murine plasma proteins were removed via ultracentrifuge filters with a cut-off of 10 kDa. The same measurements were performed in ddH_2_O as a control (data not shown).

### Cell Lines and Culture Conditions

KS (Kaposi’s sarcoma) derived from human Kaposi sarcoma [62] and HT-29 (human colorectal adenocarcinoma) cell line obtained from ATCC were cultured in RPMI 1640 medium with glutamine (Roswell Park Memorial Institute Medium, Lonza, Basel, Switzerland), and MRC-5 (normal fibroblast) cells were cultured in DMEM (Dulbecco’s Modified Eagle’s Medium, Lonza). All media were supplemented with 10% heat-inactivated FBS (Fetal Bovine Serum, Euroclone, Milan, Italy), and with 1% penicillin/streptomycin (Sigma-Aldrich). Cells were cultured in sterile T25 or T75 flasks with ventilation cap (Sarstedt, Nümbrecht, Germany) at 37 °C in a humidified atmosphere with 5% CO_2_.

### In Vitro Antiproliferative Activity of NGR-Dau Conjugates and Free Dau

For evaluation of in vitro antiproliferative activity of NGR-Dau conjugates and free Dau, cell viability was determined by MTT assay (3-(4,5-dimethylthiazol-2-yl)-2,5-diphenyl-tetrazolium bromide (Sigma Aldrich)). After standard harvesting of the cells by trypsin-EDTA (Lonza), 5 × 10^3^ till 10 × 10^3^ cells per well depending on cell line, were seeded in serum containing growth medium to 96-well plates and incubated. After 24 h, cells were treated with various concentrations of conjugate **1** and **2** (32 nM-100 μM) or free Dau (0.1-10 μM), dissolved in serum containing medium, and incubated under standard conditions. Control wells were treated with serum containing medium. Two treatment regimens were used. According to the first type, after 24 h of treatment, cells were washed with serum free medium, and then cultured in serum containing medium for an additional 48 h. In case of the second type, cells were treated for 72 h continuously. Afterward, MTT assay was performed in order to determine cell viability, by adding 20 μL of MTT solution (5 mg/mL in PBS) to each well and after 2 h of incubation at 37 °C, the supernatant was removed. The formazan crystals were dissolved in 100 μL of a 1:1 solution of DMSO (Sigma-Aldrich):EtOH (Molar Chemicals) and the absorbance was measured after 15 min at λ = 570 nm by using a microplate reader (Bio-Rad, model 550, Hercules, CA, USA). The IC_50_ values of the conjugates and free drug were calculated using GraphPad Prism 6 (GraphPad Software, San Diego, CA, USA). The experiments were done in triplicate, and each experiment was repeated two times.

### CD13 Cell Surface Expression Level Determination by Immunocytochemistry

CD-13 expression was detected by confocal microscopy. KS and HT-29 cells were seeded (10^5^ cells/well) to coverslip-containing (Assistant, Karl Hecht GmbH&Co KG, Sondheim/Rhön Germany) 24-well plates (Sarstedt) one day before immunostaining. Nuclei were stained with Hoechst 33342 solution (0.2 μg/mL, Thermo Fisher Scientific, Rockford, IL, USA, diluted in serum-free medium) for 10 min at 37 °C. After washing, cells were fixed with 4% paraformaldehyde (Sigma-Aldrich) for 20 min at 37 °C that was followed by blocking with 3% Bovine Serum Albumine (BSA, Sigma-Aldrich, dissolved in PBS) for 1 h at room temperature. Anti-CD-13 antibody (clone: WM-15, FITC-conjugated, eBioscience, 1:100, diluted in 1% BSA containing PBS) was added to the wells overnight at 4 °C. After washing three times with PBS, coverslips were mounted to cover glasses using Mowiol 4-88 (Sigma-Aldrich). Imaging was carried out using a ZEISS LSM-710 system (Carl Zeiss microscopy GmbH) with a 40x/1.4 Plan-Apochromat oil immersion objective. Images were processed with ZEN (Carl Zeiss microscopy GmbH).

### Cellular Uptake Determination by Flow Cytometry

The cellular uptake of the bioconjugates was studied on KS and HT-29 cells. Cells were seeded (1.5 × 10^5^ cells/well) to 24-well plates (Sarstedt), incubated for 24 h at 37 °C, and treated with conjugates at concentrations 2, 10, 50 and 100 μM for 6 h. After harvesting, cells were washed with PBS. Fluorescence intensity was detected using the PE-A channel of Attune NxT Flow Cytometer (Thermo Fisher Scientific). Number and proportion of the cells with intracellular fluorescence were evaluated and calculated using Attune NxT 2.6. software (Thermo Fisher Scientific).

### Experimental Animals

Adult inbred BALB/c mice from a specified pathogen free (SPF) breeding of the National Institute of Oncology (Budapest, Hungary) were used in acute and chronic toxicity studies. Mice were kept in a sterile environment in Makrolon® cages at 22-24 °C (40-50% humidity), with a lighting regulation of 12/12 h light/dark. The animals had free access to tap water and were fed with a sterilized standard diet (VRF1, autoclavable, Akronom Kft., Budapest, Hungary) *ad libitum*.

The immunodeficient SCID mice on a C.B.-17 background were bred in specific opportunistic and pathogen free isolator breeding rooms. The breeding isolator was supplied with corn-cob bedding and standard VRF1 rodent chow and with acidified (pH = 3) sterilized distilled water. The mice from the breeding rooms were used for the subcutaneous model of KS and orthotopic model of human colon cancer. They were held in filter-top boxes in the experimental barrier rooms, and every box-opening was performed under a Class 100 laminar-flow hood. The animal housing density was in accordance with the international recommendations. The cage components, corn-cob bedding and food (VRF1 from Special Diet Services) were steam-sterilized in an autoclave (121 °C, 20 min). The animals used in these studies were cared for according to the “Guiding Principles for the Care and Use of Animals” based upon the Helsinki declaration, and they were approved by the local ethical committee. Permission license for breeding and performing experiments with laboratory animals: PEI/001/1738-3/2015 and PEI/001/2574–6/2015.

### Acute and Chronic Toxicity Studies of NGR Conjugates

In order to determine toxicity of conjugates on healthy animals *in vivo*, acute and chronic toxicity studies were performed. In acute toxicity study, adult BALB/c male mice (26-32 g) were treated by a single intraperitoneal (*i.p.*) injection of both conjugates at the start of the experiment, administrating 4 different doses: 25, 12.5, 6.25 and 3.125 mg/kg Dau content (3 mice per group). In chronic toxicity studies, adult male BALB/c mice (25-31 g) were treated with both conjugates at dose of 10 mg/kg Dau content on day 1, 3, 7, 9 and 11 (5 treatments, 3 mice per group). The toxicity was evaluated on the basis of life span, behavior and appearance of the mice, as well as body weight. Parameters were followed for 14 days.

### Mouse Model of Subcutaneous Kaposi’s Sarcoma

Kaposi’s sarcoma (KS) cells were injected into SCID female mice (19-24 g) (*s.c.)*, 3 × 10^6^ cells per animal in volume of 200 μL M199 medium per animal. Treatment started 35 days after cell inoculation when average tumor volume was 66 mm^3^, by *i.p.* administration. Four groups by 5 animals were established and treated with the following doses and schedule: control group was treated with sterile water (Pharmamagist Kft., Budapest, Hungary) as solvent due to better solubility of Dau and conjugates than in saline solution; free Dau group was treated with a dose of 1 mg/kg on days 35, 42, 49, 56 and 63 after cell inoculation, while the groups treated with conjugates **1** and **2** were administered with a dose of 10 mg/kg Dau content, i.e. 33.8 and 33.5 mg/kg of each conjugate respectively, on days 35, 37, 39, 42, 45, 49, 52, 56, 59, 63 and 66 after cell inoculation. Treatment volume was 0.2 mL/animal. Animal weight and tumor volumes were measured initially when the treatment started and at periodic intervals according to the treatment schedule. A digital caliper was used to measure the longest (a) and the shortest diameter (b) of a given tumor. The tumor volume was calculated using the formula V = ab^2^ × π/6, whereby a and b represent the measured parameters (length and width). The experiment was terminated on day 70 after tumor transplantation (day 36 of treatment). The mice from all groups were sacrificed by cervical dislocation. Their primary tumors and livers were harvested and weighed.

### Mouse Model of Orthotopic Human Colon Adenocarcinoma

HT-29 human colon cancer cells were injected into SCID female mice subcutaneously (*s.c.*), 3 × 10^6^ cells per animal in volume of 200 μL M199 medium per animal, in order to establish tumor for transplantation. After 2 weeks, the mice with palpable tumors were sacrificed by cervical dislocation, and the subcutaneous tumor was dissected out aseptically. Tumor pieces of 2 mm^3^ were transplanted orthotopically, under aseptic conditions into anesthetized (narcotic mixture: tiletamine, zolazepam, xylazine, butorphanol) SCID female mice (19-25 g). A small midline incision (0.5 cm) was made and the colorectal part of the intestine was exteriorized. Serosa of the site where the tumor pieces were to be implanted were removed. Tumor tissue fragments of HT-29 human colon tumor were implanted on the top of the animal intestine; an 8/0 surgical (polypropylene) suture was used to suture it on the wall of the intestine. The intestine was returned to the abdominal cavity, and the abdominal wall was closed with 4/0 surgical (polyglycolic acid) sutures. The wound was sterilized and the animals were kept in a sterile environment. On the next day, no sign of pain and/or stress of the mice was observed. The treatments started 6 days after tumor transplantation by *i.p.* administration of the compounds dissolved in distilled water for injection. 8 mice per group were used.

One group of mice were treated with free Dau (1 mg/kg body weight) on days 6, 13 and 20 after tumor transplantation. Animal groups treated with compounds **1** and **2** were administered with a dose of 10 mg/kg Dau content (33.8 and 33.5 mg/kg of each conjugate, respectively) on days 6, 8, 10, 13, 17, 20 and 24 after tumor transplantation. Control group was treated with sterile water as solvent in 0.2 mL volume per animal. The experiment was terminated on day 27 after tumor transplantation (day 22 of treatment). Daunorubicin treated group was terminated on day 24 after tumor transplantation (day 19 of treatment) due to significant weight loss of the animals. The mice from all groups were sacrificed by cervical dislocation. Their primary tumors and livers were harvested and weighed, while metastases were counted in other organs.

### Determination of the Proliferative Index and Vascularization in Tumor Tissues

The routinely formalin-fixed tumors were dehydrated in a graded series of ethanol, infiltrated with xylene and embedded into paraffin at a temperature not exceeding 60 °C. Two microns thick sections were mounted on Superfrost slides (Thermo Shandon, Runcorn, UK) and manually deparaffinized. To block endogenous peroxidase activity, slides were treated for 20 min at RT with 3% H_2_O_2_ in methanol. Slides were immersed in 6% citrate buffer (pH = 6) and exposed to 98 °C water bath for 40 min. Afterwards, slides were primarily treated with antibody against human KI-67 (DAKO, Glostrup, Denmark, 1:40) and endothelial marker CD31 (Dianova, Hamburg, Germany, 1:20) incubated for 1 h at RT. After washing, Biotinylated Link (Dako) secondary antibody was applied for KI-67 samples for 10 min at RT, while rabbit anti-rat IgG (Novus Biologicals, Centennial, CO, USA) was applied for CD31 samples for 1 h at RT. For visualization of KI-67 samples, supersensitive one step polymer HRP (Biogenex, Fremont, CA, USA) was used with 3-amino-9-ethylcarbazole (AEC) as chromogen, while for visualization of CD31 samples match 2 rabbit-HRP polymer (Biocare Medical, Concord, CA, USA) with AEC (Vectorlabs, Burlingame, CA, USA) were used. Staining without the primary antibody served as negative control. The KI-67-positive tumor cells were counted manually per fields of vision under light microscope (400-fold magnification), and 3 fields of view per tumor were evaluated. Proliferation index was calculated as percentage of KI-67 positive cells from all cells in the field of view. The CD31-positive blood vessels were counted manually using light microscope (200-fold magnification), whereby 3 fields of view per tumor were evaluated, and blood vessel density was calculated as number of blood vessels per mm^2^.

### Statistical Analysis

The statistical analyses were performed by GraphPad Prism 6 (GraphPad Software) using the non-parametric Mann-Whitney (independent samples) test. The experimental data were filtered by Gaussian statistics where *P*-values lower than 0.05 were considered statistically significant.

## Results

### In Vitro Antiproliferative Activity of NGR-Dau Conjugates and Free Dau

The antiproliferative effect of the NGR-Dau conjugates **1** and **2** and free Dau was investigated in vitro on CD13(+) Kaposi’s sarcoma cells (KS) and on CD13(−), but integrin positive [63] HT-29 human colorectal adenocarcinoma cells, as well as on MRC-5 (human fibroblast) as non-cancerous control cell line.

**Fig. 2:**
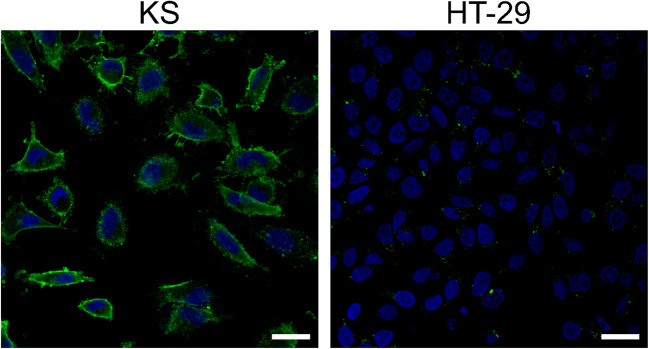
Determination of cell surface CD13 expression on KS (left) and HT-29 cells (right) by immunocytochemistry. CD13 was detected by anti-CD13-FITC antibody (green). The nuclei were stained with Hoechst 33342 (blue). The scale bars represent 20 μm

Before performing antiproliferative activity studies, cell surface CD13 expression of KS and HT-29 cells was detected by immunocytochemistry and visualized by confocal microscopy. As stated in the literature, KS cells express a higher level of CD13 receptors at their cell membrane compared to HT-29 cells (Fig. 2).

The results of the MTT assay showed that both conjugates have antiproliferative effect on cancer cells (Table 1). Conjugate **2** displayed higher antiproliferative activity than conjugate **1**. The conjugates showed higher antiproliferative activity on CD13(+) KS cells, then on CD13(−) HT-29 cells. The IC_50_ values of both conjugates are lower after longer time of exposure, particularly for conjugate **2**. High IC_50_ values of conjugates were obtained on MRC-5 cells, showing selectivity of conjugates for cancer cell lines, especially in case of conjugate **1**.

**Table 1:** In vitro antiproliferative activity of NGR-Dau conjugates and Dau on KS, HT-29 and MRC-5 cells after 24 h treatment followed by 48 h incubation in drug free medium and 72 h continuous treatment. All IC_50_ values represent average ± SD

	KS (24 h) IC_50_ (μM)	KS (72 h) IC_50_ (μM)	HT-29 (24 h) IC_50_ (μM)	HT-29 (72 h) IC_50_ (μM)	MRC-5 (72 h) IC_50_ (μM)
Dau	0.1 ± 0.01	0.07 ± 0,01	0.09 ± 0.01	0.1 ± 0.01	0.25 ± 0.06
1	22.7 ± 2.3	15.2 ± 0.3	32.4 ± 1.4	29.3 ± 1.0	> 100
2	8.0 ± 0.8	4.3 ± 0.1	13.9 ± 1.6	7.2 ± 0.2	25.4 ± 3.5

### Determination of Cellular Uptake of NGR-Dau Conjugates

**Fig. 3:**
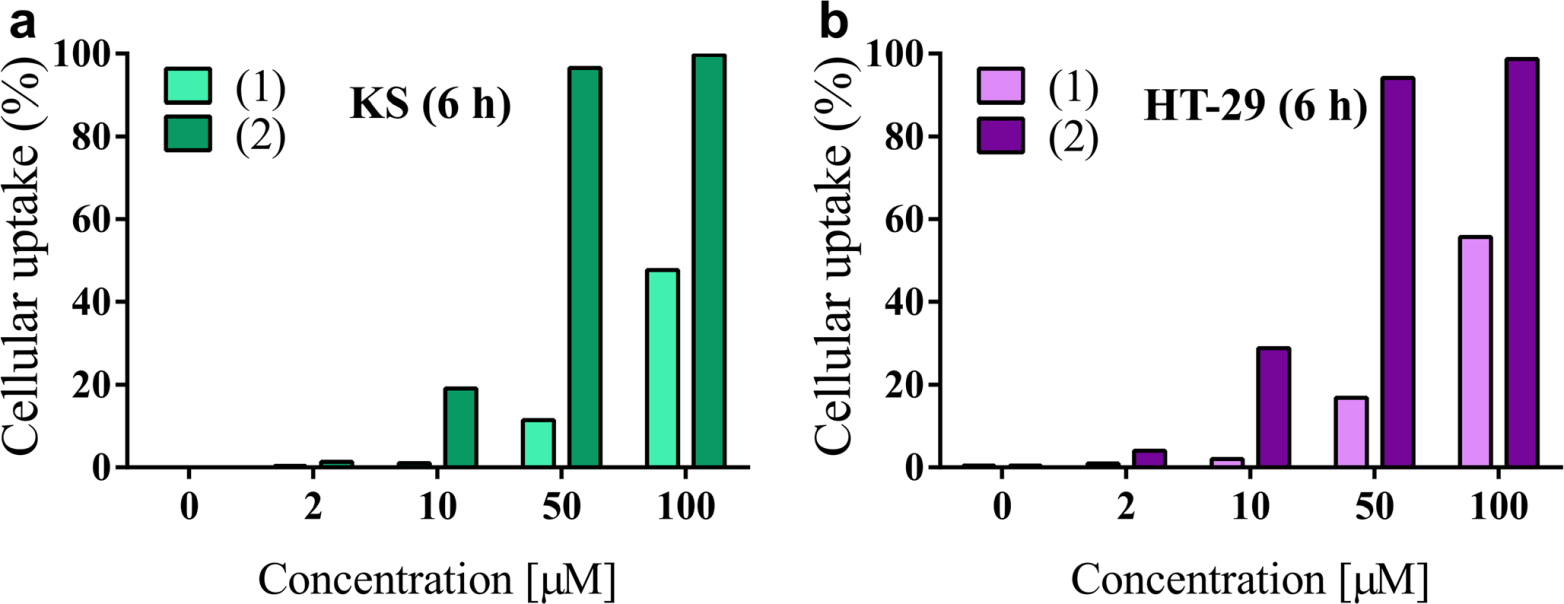
Cellular uptake of NGR-Dau conjugates **1** (Lys containing) and **2** (Nle containing) at concentrations 2, 10, 50 and 100 μM, after 6 h treatment by KS cells (**a**) and HT-29 cells (**b**)

The cellular uptake of the NGR-Dau conjugates by KS and HT-29 cells was measured by flow cytometry. The obtained results displayed that the uptake on both cell lines was concentration dependent, but the new conjugate **2** entered the cells more efficiently than **1**, especially at lower concentrations (Fig. 3a,b). At 50 μM concentration, conjugate **2** showed almost 100% uptake, whereas conjugate **1** was lower (~20%). At 100 μM concentration, conjugate **1** was already taken up by around 50% of cells. Moreover, at 50 μM and 100 μM concentrations, the uptaken level of conjugate **2** by CD13(+) KS cells was similar to those in HT-29 cells, while for conjugate **1** higher values were measured in case of HT-29 cells.

### Stability in Mouse Plasma

The stability of the two bioconjugates was determined in murine plasma, using HPLC-MS. Both conjugates were stable at experiment conditions for at least 8 h at 37 °C. These findings might be relevant and promising if we consider their importance for *in vivo* applications.

### Acute and Chronic Toxicity Studies of NGR-Dau Conjugates

**Fig. 4:**
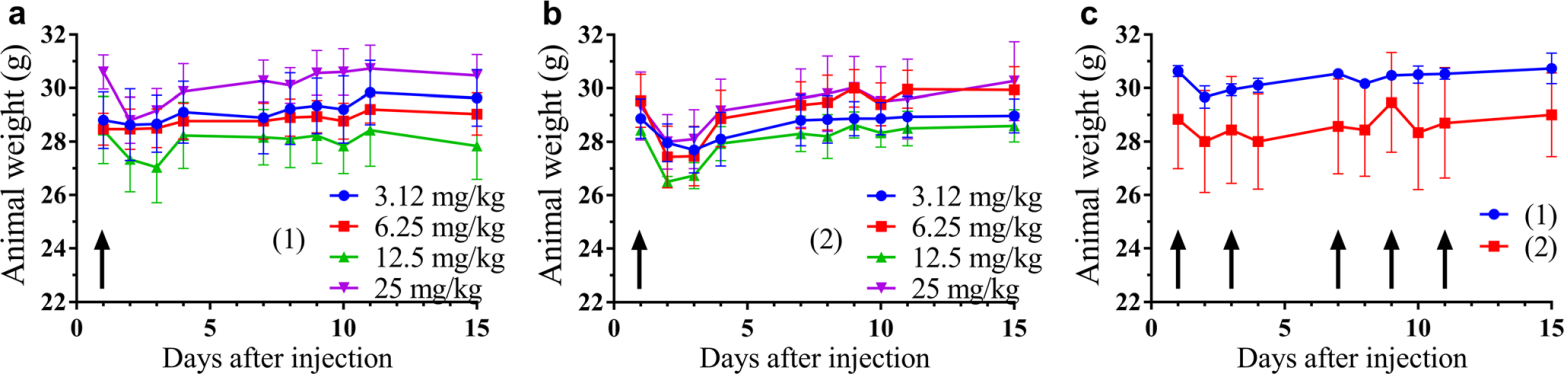
Animal body weight (g, average ± SEM). **a** Acute toxicity study of conjugate **1** with doses of 3.125, 6.25, 12.5 and 25 mg/kg Dau content. **b** Acute toxicity study of conjugate **2** with doses of 3.125, 6.25, 12.5 and 25 mg/kg Dau content. **c** Chronic toxicity study of NGR-Dau conjugates **1** and **2** with dose of 10 mg/kg Dau content, 5 treatments: marked by black arrows. 3 mice per group

Acute toxicity experiment was performed for 14 days in adult male BALB/c mice. No significant change in body weight could be observed **(**Fig. 4a,b), and also the general looking and behavior of experimental animals were adequate, even when 25 mg/kg Dau content of both NGR-Dau conjugates was used. Chronic toxicity experiment was also performed for 14 days, and animals were treated with both conjugates 5 times at a dose of 10 mg/kg Dau content. Similarly to the acute toxicity experiment, we could not observe a significant change in body weight (Fig. 4c), general looking and behavior of the mice.

### Effect of NGR-Dau Conjugates and Free Dau in Kaposi’s Sarcoma Subcutaneous Model *in Vivo*

**Fig. 5:**
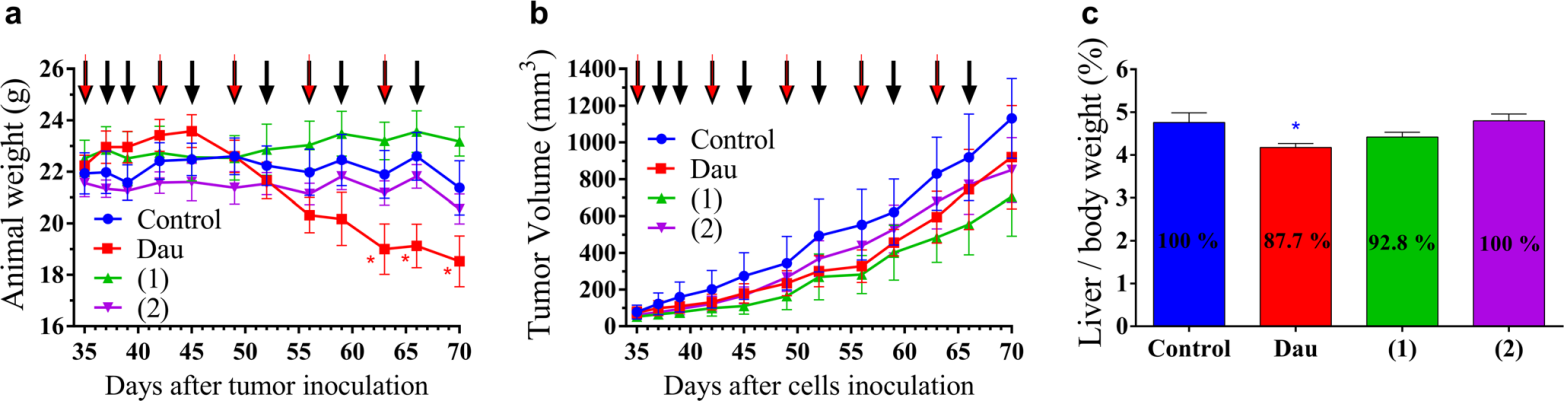
Effect of NGR-Dau conjugates **1** and **2** (10 mg/kg Dau content, 11 treatments, black arrows) and free Dau (1 mg/kg, 5 treatments, red arrows) on subcutaneous Kaposi’s sarcoma bearing mice. **a** Animal body weight (g, average ± SEM). **b** Tumor volume (mm^3^, average ± SEM). **c** Liver/body weight ratio (percentage, average ± SEM) after termination of experiment, 70 days after cell inoculation, 5 animals per group. Statistical analysis was performed by Mann-Whitney test. * means significantly different at *p* < 0.05

Subcutaneous Kaposi’s sarcoma bearing SCID mice were treated with NGR-Dau conjugates **1** and **2** at dose of 10 mg/kg Dau content 3 times during the first week and two times per week during the next 4 weeks, as well as with free Dau at a dose of 1 mg/kg once per week, and their effect on the animal body weight was evaluated (Fig. 5a). The animal body weight was decreased non-significantly in the control and conjugate **2** treated groups (2.5% and 4.6%, respectively), while non-significant increase was obtained in conjugate **1** treated group (3%). In comparison, administration of free Dau from day 63 after cell inoculation resulted in a significant decrease of mice body weight which was at the end of experiment reduced by 16.7%. Considering that some animals of the control group and all animals in the free Dau treated group were in bad condition, the experiment was terminated on day 70 after cell inoculation.

The antitumor effect of NGR-Dau conjugates **1** and **2** and free Dau was evaluated by measuring the tumor volume in each group. All treated groups showed decreased tumor volume in comparison to control group at the end of the experiment (Fig. 5b). Treatment with conjugate **1** was the most effective, whereby the tumor volume was inhibited by 37.7% compared to the non-treated control. Conjugate **2** inhibited tumor growth by 24.8%, while the group treated with free Dau showed the lowest inhibition of tumor volume, only 18.6% compared to control.

The effect of NGR-Dau conjugates and free Dau on liver toxicity was evaluated by measuring the liver weight at the end of the experiment and calculating the liver weight/body weight ratio (Fig. 5c). The average liver/body weight ratio from mice in the group treated with free Dau decreased significantly (*p* < 0.05) by 12.3% in comparison to control group. The groups treated with conjugates **1** and **2** showed no significant changes in liver/body weight ratio in comparison to the control (92.8% and 100%, respectively.

### Effect of NGR-Dau Conjugates and Free Dau in Orthotopic HT-29 Human Colon Tumor Model *in Vivo*

**Fig. 6:**
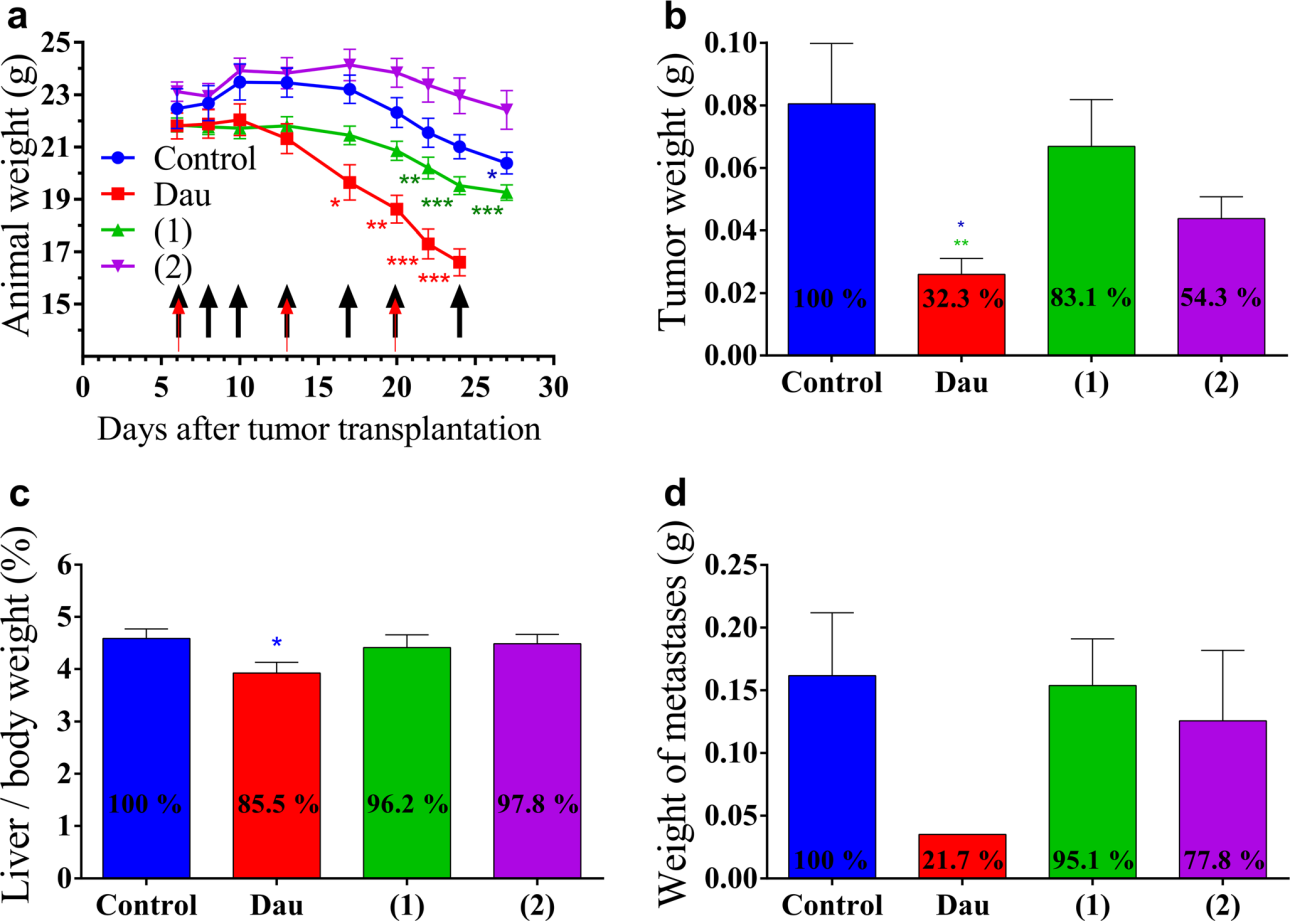
Effect of NGR-Dau conjugates **1** and **2** (10 mg/kg Dau content, 7 treatments, black arrows) and free Dau (1 mg/kg, 3 treatments, red arrows) on orthotopic HT-29 human colon carcinoma bearing mice. **a** Animal body weight (g, average ± SEM). **b** Tumor weight (g, average ± SEM). **c** Liver/body weight ratio (percentage, average ± SEM). **d** Weight of metastases near the primary tumor (g, average ± SEM). Control and groups **1** and **2** were measured after the termination of the experiment (on day 27 after transplantation), while Dau group was measured on day 24 subsequent to transplantation. 8 animals were used per group. Statistical analysis was performed by Mann-Whitney test. *, ** and *** mean significantly different at *p* < 0.05, *p* < 0.01, and *p* < 0.001, respectively

Orthotopic HT-29 human colon carcinoma bearing SCID mice were treated according to the same doses and schedule as subcutaneous Kaposi’s sarcoma bearing mice. The animal body weight decreased in all groups at the end of the experiment compared to the start (Fig. 6a). The mice treated with free Dau exhibited a significantly decreased body weight from day 17 after tumor transplantation, whereby the experiment was terminated on day 24 (day 19 of treatment). Conjugate **1** caused significant decrease in body weight from day 22, while the body weight of the control group significantly decreased on the last day of the experiment and due to this the experiment was terminated on day 27 after tumor transplantation. No significant change in the body weight was obtained during the treatment period with conjugate **2**.

The antitumor effect of the NGR-Dau conjugates and free Dau was evaluated by measuring the tumor weight in each group after termination (Fig. 6b). The obtained data reveal that the free drug inhibited the tumor growth significantly, whereby the tumor weight was reduced by 67.7% compared to the control group. Conjugate (**1**) inhibited tumor growth by 16.9%, while inhibition by conjugate **2** was 45.7% in comparison to the control group.

The toxicity of the free drug was detected in this experiment as well. The average liver/body weight ratio of the mice treated with free Dau was significantly (*p* < 0.05) decreased by 14.5% in comparison to the control group (Fig. 6c). In contrast, there was no significant change in liver/body weight ratio in the groups treated with the conjugates (96.2% and 97.8% of the control, respectively). The antimetastatic effect of NGR-Dau conjugates was evaluated by counting and measuring the weight of metastases near the primary tumor in each group at the end of the experiment (Fig. 6d). Lower number of metastases was observed near the primary tumor in the treated groups compared with the untreated control group. While in control group metastases were found in 7 out of 8 animals, in treated groups using Dau, conjugates **1** and **2,** metastases were found in 1, 5 and 4 out of 8 animals, respectively. Free Dau inhibited the weight of metastases by 78.3%. Conjugate **1** did not show significant inhibition (4.9%), while conjugate **2** inhibited metastases by 22.2% in comparison to control group.

### Effect of NGR-Dau Conjugates and Free Dau on Proliferation and Vascularization in Primary Tumor

**Fig. 7:**
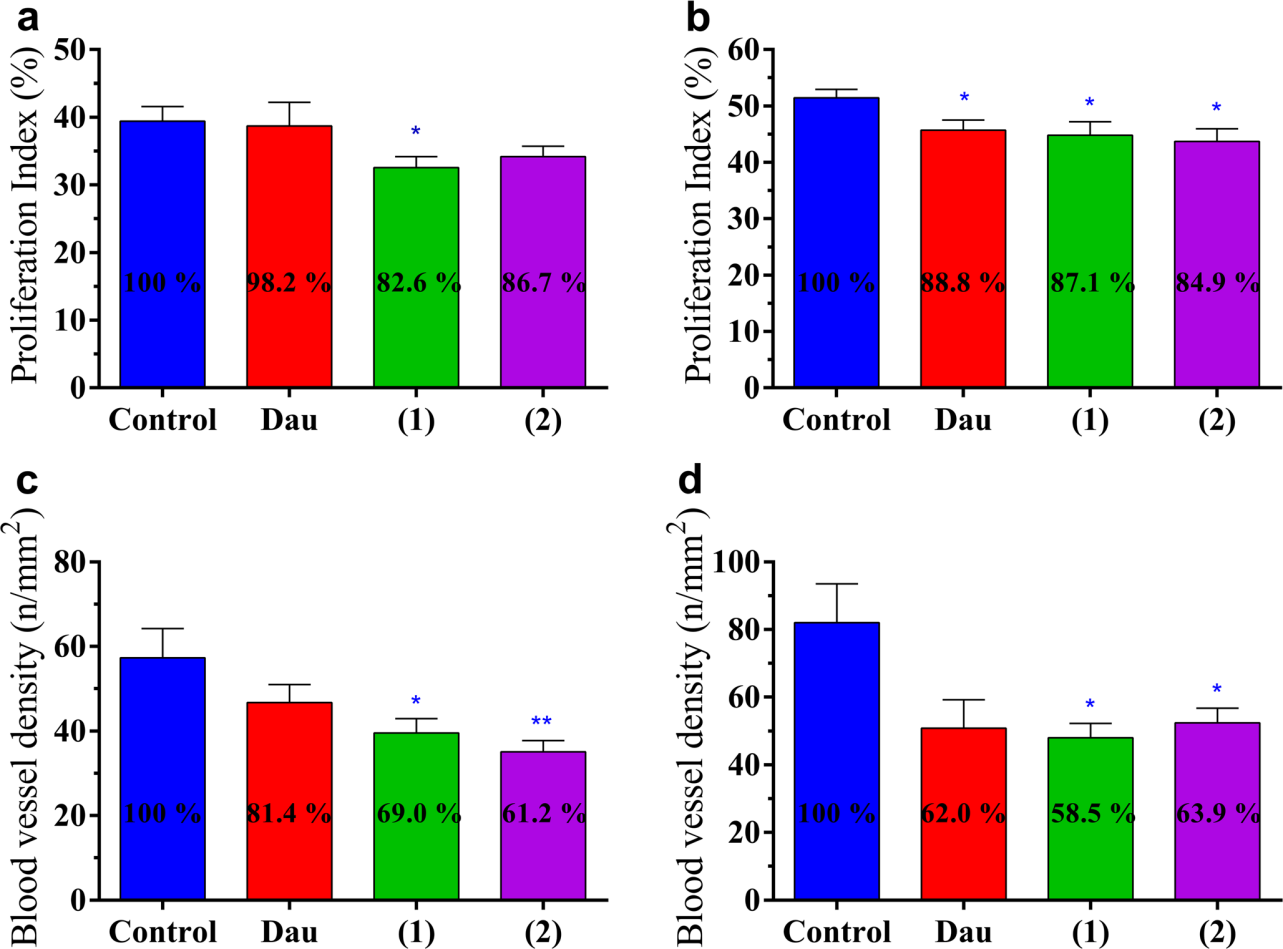
Effect of NGR-Dau conjugates **1** and **2** and free Dau on proliferation in primary tumor in Kaposi’s sarcoma (**a**) and HT-29 human colon carcinoma (**b**) bearing mice. Effect of NGR-Dau conjugates **1** and **2** and free Dau on vascularization in primary tumor in Kaposi’s sarcoma (**c**) and HT-29 human colon carcinoma (**d**) bearing mice. Values represent average ± SEM. Statistical analysis was performed by Mann-Whitney test. * and ** mean significant at *p* < 0.05 and *p* < 0.01, respectively

The effect of NGR-Dau conjugates and free Dau on proliferation was evaluated in the primary tumor by proliferation index, presented by the percentage of proliferation marker (KI-67) positive cells out of all cells per field of view in subcutaneous Kaposi’s sarcoma and orthotopic HT-29 human colon primary tumors (Fig. 7a,b). In KS primary tumor, all treated groups showed decreased proliferation, but only the group treated with conjugate **1** displayed significant inhibition of proliferation by 17.4% compared to the control. However, in HT-29 primary tumor, all treated groups showed significant inhibition of proliferation by 11.2, 12.9 and 15.1% for Dau, and conjugates **1** and **2**, respectively.

The effect of NGR-Dau conjugates and free Dau on vascularization in the primary tumor was evaluated by counting CD31 (endothelial marker) stained blood vessels per field of view in subcutaneous Kaposi’s sarcoma and orthotopic HT-29 human colon primary tumors, and by determining blood vessel density and number of blood vessels per mm^2^ (Fig. 7c,d). In KS primary tumor, all treated groups showed lower blood vessel formation, but we could detect significant inhibition only in groups treated with conjugates: 31.0% (**1**) and 38.8% (**2**) compared to the control. In HT-29 primary tumor, all treated groups showed inhibition of vascularization which was significant only when treated with conjugates **1** and **2** by 41.5% and 36.1%, respectively (whereas for Dau-treated group the calculated *p* value was 0.0671 in comparison with the control group).

## Discussion

Kaposi sarcoma, first described by dermatologist Moritz Kaposi in 1872 is a tumor of vascular origin occurring in many different clinical-epidemiological forms with several lesions of the skin [60, 64, 65]. This type of angio-proliferative disease nowadays represents one of the most aggressive type of tumors in HIV-1-infected individuals [66]. It was demonstrated that primary lesions of KS express a very high level of CD13/APN and this contributes to the enhanced vascularization of the tumor [22, 67]. Our attention in the present study is also focused on a colon carcinoma tumor model which is one of the most lethal forms of tumor in developed countries. Colon carcinoma cells can express a variety of integrins but only a lower level of CD13 [63, 68]. Targeted tumor therapy is a promising approach to reduce all the disadvantages of chemotherapeutic agents [4]. In the present study, cyclic NGR-Dau peptides were used as targeting moieties due to their affinity to CD13 and a variety of integrins, in particular the ones strictly connected to angiogenesis (RGD integrins). The recognition of integrins by NGR peptides is rather based on the rearrangement of NGR to *iso*DGR peptides developed by succinimide formation followed by hydrolysis. *Iso*DGR peptides similarly to RGD ones can efficiently recognize different integrins like α_v_β_3_, α_v_β_6_, etc. [55, 56, 69] The importance of these homing devices relies on the fact that they have their own in vitro antiproliferative effect. Our recently published data indicated that cyclic NGR peptide with daunorubicin connected via chemo selective ligation (oxime bond formation) can have significant antitumor activity in vitro. In this study, the antitumor effect of ours two best conjugates in comparison with the free drug was examined in vitro on CD13(+) KS and CD13(−) HT-29 cells, while MRC-5 normal fibroblasts were used as negative control. While the free daunorubicin is uptaken rather via diffusion, the conjugates are ideally internalized through receptor-mediated endocytosis leading to the release of the active metabolite in lysosomes. In our case it is Dau = Aoa-Gly-OH that can bind to the DNA resulting in cell death [70]. The results of in vitro MTT assay showed that both conjugates have higher antitumor effect on KS (CD13+) than HT-29 (CD13-) cells. In addition, conjugate **2** with Nle instead of Lys in the cycle provided significantly higher efficiency on both cell lines, which is supported by cellular uptake studies where conjugate **2** entered the cells more efficiently than **1**, especially at lower concentrations. The increased antitumor effect might be explained by the faster rearrangement from NGR to *iso*DGR in case of conjugate **2** in comparison with conjugate **1** resulting in dual acting propensity of the conjugate [57]. The cytotoxicity of conjugates in MRC-5 cells was fairly low compared to the tumor cells. The selectivity towards cancer cells was especially high in case of conjugate **1** that is more stable against deamidation.

Taking into account the obtained results, it can be proposed that the biological activity of the NGR-Dau conjugates depends strongly on the expression of CD13 receptor which ensures the selectivity of the conjugates and their cellular uptake capacity.

Due to the promising in vitro results with conjugates **1** and **2**, we decided to analyze their *in vivo* antitumor activity on tumor-bearing mice. Initially, we determined the stability of the compounds in mice plasma detecting that both conjugates were stable for 8 h [37, 57].

For the drug development process, the studies of the toxicity effect of new compounds on healthy mice is essential. Thus, acute toxicity study was performed, showing that neither the body weights of mice, nor the general looking and behavior of experimental animals were significantly changed, even at a dose of 25 mg/kg Dau content of both conjugates. To further evaluate the effect of the compounds on healthy mice, chronic toxicity was investigated where animals were treated with both conjugates 5 times with a dose of 10 mg/kg Dau content. After 14 days, we could not observe any significant change in the body weights, general looking and behavior of mice. Based on these results, it could be concluded that both conjugates were suitable for treatment of tumor bearing mice at this concentration.

First, the *in vivo* antitumor activity of NGR-Dau conjugates and free Dau was determined on a subcutaneous KS model. The animal body weight of mice indicated significant decrease for the Dau group, while in control and in conjugates treated groups, no significant change in animal body weight could be observed indicating that the conjugates did not cause toxic side effects to the animals during the treatment in comparison with the free drug.

The antitumor effect of the NGR-Dau conjugates **1** and **2**, as well as the free Dau was evaluated by measuring the tumor volume in each group during the experiment. We obtained tumor growth inhibition of KS tumor in all treated groups compared to the control group. Interestingly, in contrast to the in vitro experiments, the inhibition effect of conjugate **1** was higher in comparison with Dau and conjugate **2**. Data showed anti-tumor effect of the conjugates against KS tumor, especially in case of conjugate **1** which inhibited tumor volume by 37.7% compared to control.

The number of dividing cells in tumor tissues, as well as the formation of blood vessels are strictly associated with cell proliferation and tumor progression. Therefore, the proliferation index of KI-67 positive cells and blood vessel density by CD31 marker in the primary tumor were determined [71, 72]. Both the cell proliferation index and blood vessel formation were lower in case of mice treated with conjugates in comparison with the Dau-treated and control group. The differences were higher in case of conjugate **1** than in case of **2**. The results can explain the higher tumor growth inhibition of conjugate **1.** We can also conclude that for the treatment of a highly CD13 positive tumor type, a stable NGR peptide derived conjugate might be a better choice.

Furthermore, not only the elicited antitumor activity is of high relevance for the success of an anticancer drug, but also the selectivity to cancerous cells and reduction of side effects, thus, liver toxicity was determined. Since the liver is the vital organ in drug metabolism, this analysis provides a better understanding of drug toxicity [73]. Free Dau caused a significant decrease of liver/body weight ratio in comparison to control group, revealing that treatment with Dau resulted in toxic side effect in mice. In comparison, non-significant liver/body weight ratio changes could be detected in NGR-Dau conjugates treated groups proving evidences for their selectivity and non-toxicity to healthy tissue.

Next to the *in vivo* studies on KS bearing mice, we were interested in analyzing the anticancer activity of the two NGR-Dau conjugates and free Dau on a tumor that has a lower expression of CD13 receptors but expresses integrin receptors [66].

Regarding our evaluation of CD13 receptor expression and based on studies that pointed out that HT-29 cell line shows increased integrin receptor expression level, this human colon adenocarcinoma might represent an adequate model for this experiment [58]. Although orthotopic colon cancer xenograft models are technically challenging and labor-intensive, orthotopic transplants are able to mimic human tumors more accurately. This approach simulates the natural microenvironment for tumor development better, providing an effective approach to investigate tumor pathophysiology and to develop therapeutic strategies which allow a better prediction of patient’s response to chemotherapy in comparison with heterotopic transplants [74]. A variety of synthetic therapeutics have been used which target receptors, and hence revealed a significant tumor growth inhibition in vitro and *in vivo*. Thus, HT-29 human colon tumors were implanted to the intestine of immunodeficient SCID mice [75–77].

We observed that the free Dau caused a significant decrease in mice body weights which compelled us to terminate the experiment for this group on day 24 after tumor transplantation. For similar reasons, conjugate **1** treated group was also terminated on day 27. Significant changes of animal weight were not observed in case of mice treated with conjugate **2** (terminated also on day 27). This indicates that both conjugates induce less harmful side effects than Dau, especially conjugate **2** with no significant effect. Moreover, it might be possible that the decrease in body weight was caused by a higher susceptibility of the immunodeficient animals after surgery procedures which was necessary to establish the orthotopic colon cancer model [78].

The antitumor effect of the conjugates and Dau was evaluated after isolation of the tumors at the end of the experiment [79]. We obtained a significant inhibition of the tumor weight only in Dau treated group compared to the control, which showed significant inhibition compared to conjugate **1** also. High inhibition of tumor weight (45.7%) was obtained in the treatment with conjugate **2**, though the decrease was not significant. The better efficacy of conjugate **2** on orthotopically developed CD13(−) HT-29 colon cancer might be explained by the faster rearrangement of conjugate **2** to *iso*DGR derivative that recognizes integrins in comparison with conjugate **1**.

Evaluation of the proliferation index in primary tumors revealed that both conjugates and free Dau as well decrease the proliferation rate supporting their significant inhibitory effect in primary tumors. This can also be supported by the significant inhibition of new blood vessel formation by both NGR-Dau conjugates revealing their suppressing effect on angiogenesis in HT-29 primary tumor. Higher inhibition of vascular density of conjugate **2** in KS tumors compared to colon tumors can be explained also by the faster rearrangement of conjugate **2** to *iso*DGR derivative that recognizes integrins. As KS cells express both α_v_β_3_ and α_v_β_5_ integrins [64] compared to HT-29 cells expressing only α_v_β_5_ integrin [58], we can assume that the dual targeting activity is more pronounced in case of KS tumor. The effect of conjugates on blood vessel density was higher than on proliferation index. Functional antagonists of CD13/APN inhibit capillary tube formation [22] interfering with highly expressed CD13 on intratumoral blood vessels [10, 16, 25, 26], suppressing the nutrient supply necessary for tumor cell viability and tumor proliferation which inhibition occurs in later stages [35, 80].

It has been reported that in SCID mice, the remaining innate immune cells reduce the metastasis formation in distal organs [81]. This can be a possible reason for the lack of metastases in peripheral organs in our study. Consequently, the antimetastatic effect of the conjugates and free drug was evaluated based on the number of animals containing metastases close to the primary tumor and also on the total weight of metastases. The obtained results showed that the antimetastatic effect of Dau was the highest with only 1 animal with metastases close to the primary tumor, while conjugate **2** showed better antimetastatic effect than conjugate **1** (4 and 5 animals with metastases close to the primary tumor, respectively) which reduced total metastasis weight by 22% compared to control where 7 out of 8 animals were with metastases suggesting that these two conjugates has potential antimetastatic therapeutic effect for colon cancer.

In addition, we did not detect significant changes in the liver/body weight ratio of the groups treated with the conjugates, while a significant decrease was observed for the group which was treated with the free drug. This indicates that the conjugates did not cause toxicity in mice, unlike free Dau.

Based on these results, we can conclude that both NGR-Dau conjugates, as well as free Dau inhibited tumor growth and metastasis development. However, conjugate **2** showed higher antitumor and antimetastatic effect against colon cancer compared to conjugate **1**, while its impact on the animal body weight and liver weight was the lowest. Both conjugates demonstrate significant effect on inhibition of proliferation in the primary tumor and inhibition of blood vessels formation making them promising candidates for targeting angiogenesis processes in tumor tissues.
